# New proposed ITER divertor design using carbon insert on tungsten to mitigate ELMs and secondary radiation effects on nearby components

**DOI:** 10.1038/s41598-022-08837-2

**Published:** 2022-03-18

**Authors:** V. Sizyuk, A. Hassanein

**Affiliations:** Center for Materials Under Extreme Environment (CMUXE), Purdue University, West Lafayette, IN 47907 USA

**Keywords:** Nuclear fusion and fission, Computational methods

## Abstract

Building a successful device for the magnetic fusion energy production is a great challenge. ITER is an international project of the tokamak based magnetic fusion design being developed for the demonstration of the feasibility of thermonuclear technologies for future realization of successful commercial fusion energy. A key obstacle to a successful magnetic fusion energy production is however, the performance during abnormal events including plasma disruptions and edge-localized modes (ELMs). A credible reactor design must tolerate at least a few of these transient events without serious consequences such as melting of the structure. This paper investigates and compares the performance of the current ITER tokamak design during two types of transient events, i.e., ELMs occurring at normal operation and disruptions during abnormal operation. We simulated the divertor components response using our integrated 3D HEIGHTS package. The simulations include self-consistent modeling of the interaction of the released core plasma particles with the initial solid divertor material, energy deposition processes, vaporization of divertor material, secondary plasma formation and MHD evolution, incident core particles collisions and scattering from this dense secondary plasma, photon radiation of secondary plasma, and the resulting heat loads on nearby components. Our simulations showed that using a small carbon insert around the strike point can significantly reduce the overall expected damage on the tungsten dome structure, reflector plates, and prevent tungsten vaporization and its potential core plasma contamination.

## Introduction

The International Thermonuclear Experimental Reactor (ITER) is currently being built in France to demonstrate the feasibility of magnetic fusion for energy production. Many physical processes should be integrated self-consistently during to correctly assess ITER and future tokamak successful performance. Currently, these phenomena cannot be completely studied experimentally in existing tokamak devices due to the huge power density projected in future devices. To correctly predict future tokamak operation during abnormal and transient events, researchers should generally extrapolate current experimental data from existing tokamaks^1^ or simulate many occurring processes and physical phenomena self-consistently in real 3D device geometry with precise details, i.e., with comprehensive end-to-end all-inclusive model^2^.

However, new impediments and challenges rise due to the large increase in power and size of fusion devices. In particular, plasma material interactions and resulting performance of plasma facing components (PFC) are the main restraints for a constructive magnetic fusion device in regard to transient events, i.e., edge-localized modes (ELMs) at normal operation and disruptions during abnormal operation. During a disruption, the entire core plasma energy will be lost at once due to such effects as MHD instabilities while about 10% of the plasma energy is lost due to an ELM event occurring at normal operations.

While major efforts are being examined to find confident and robust ways to predict and mitigate such events, a vigorous reactor design must be able to tolerate without significant damage a few of these transient events, both during normal and abnormal operations. Development of successful fusion reactors critically depends on the correct prediction of the heat and particle loads to various reactor components and identifies the optimum materials choice for PFC. Currently, there are several powerful computer codes developed for simulation of the core-SOL plasma behavior and prediction of divertor power exhaust during normal and transient operations in ITER-like devices^3,4^. These codes are focused on accurate modeling of the core hydrogen isotopes plasma evolution with perturbation due to wall material impurities. This approach is ideal for simulation of the tokamak normal operation. However during the transient events, the divertor space is immediately filled with a dense secondary plasma of the divertor material due to the intense heat and particle loads around the strike point (SP). Such detail calculations predicted a massive flow of this secondary plasma with density up to ~ 10^17^ cm^−3^ near the divertor plate; that is much higher than the SOL D/T plasma density ~ 10^13^ cm^−3^ by several orders of magnitude. As a result, the near-surface dense divertor-produced plasma evolution and its MHD is the main driving force while the incident D/T hot plasma is considered as a small perturbation due to the large difference in the density of these two plasmas. We focused our study on the detail production of the secondary plasma generation, evolution, propagation upward through the SOL and its interaction with the disrupting hydrogen plasma, and their effects on divertor component and nearby surfaces. As far as we know, HEIGHTS package is the only package that combines and integrates all these interaction processes in actual ITER full 3D geometry including the complex magnetic fields structure.

During transient events, the escaping core plasma initially striking the divertor plate around the SP. The escaping particles are generally varying in space and time and will cause vaporization of the divertor material, ionizing such material forming a denser secondary plasma, and then continue to interact with this evolving secondary divertor plasma during the course of the transient ELMs and disruptions. We have developed comprehensive multidimensional models for the integrated simulation of the escaped core plasma particles flow, detail energy deposition, and interaction with the divertor and nearby components. The evolution of the escaped core plasma particles is simulated in 3D realistic ITER geometry in the presence of both the complex magnetic and electric fields starting from the last closed flux surface (LCFS) through the entire scrape-off layer (SOL) area and through the penetration depths inside the divertor components^5,6^.

The objective of this work is to accurately and comprehensively simulate the generated secondary plasma evolution, propagation, and interactions with various reactor components following a disruption or an ELM event originally incident at the tungsten or carbon SP locations. Contrarily to the core DT rare plasma, the secondary dense plasma has completely different physics and hydrodynamic evolution. This generated plasma of the divertor material and its dynamic evolution in the strong magnetic fields will determine the energy transport of the core plasma in the divertor space and the damage of the divertor plates and nearby components. Despite disruptions and ELMs have quite different characteristics, both have major concerns regarding the performance of ITER and future designs. We used our upgraded HEIGHTS integrated 3D simulation package^5^ and focused the present study to analyze transient events of ELMs and disruptions of 1 ms duration as most probable for the current ITER design^7–9^. We compared the current tungsten divertor design with our proposed tungsten–carbon design with carbon only covering a small strip area around the SP surrounded by the tungsten. Therefore, tungsten (high-Z) is covering most of the divertor plate and carbon strip (low-Z) is only covering small area around the SP where most of the disrupting energy deposit their energy. This is mainly to take advantage of the much lower radiation power of the carbon secondary plasma generated compared to the tungsten plasma which shown to cause significant damage to nearby and hidden internal components that are difficult to repair without significant downtime of the reactor.

## Integrated model requirement

The current scaling rules of plasma energy deposition and prediction of plasma-facing components (PFC) response worked well for small tokamaks without significant complexity or overlap of these processes. However, this situation changes considerably when increasing the tokamak power to a level causing appreciable or significant erosion during such transient events. The initial heat loads on the divertor plates from ELM or disruption transient events will melt and vaporize the divertor material. The flow of energy transport from the escaping core plasma particles continues to heat and ionize the vaporized material and produces a secondary divertor plasma that expands into the scrape-off layer (SOL) and interacts with the incoming DT fuel plasma^5^. The simple extrapolation methods to evaluate the damage from the transient events are no longer valid due to the involvement of a new phenomenon, i.e., the secondary dense divertor-generated plasma formed from the vaporized divertor materials. The SOL domain undergoes significant changes in properties and behavior after the expansion of the secondary plasma and its interaction with the incoming main or disrupting DT plasma. It is the complex detail properties of the divertor plasma generation, motion, and its dynamic interaction with the incoming core plasma particles that will determine the overall resulting damage and consequences from such events. As a result, the earlier evaluations regarding various potential divertor materials (carbon, tungsten, liquid metals) didn’t include such consequences of their response to transient events on the overall resulting damage.

**Figure 1:**
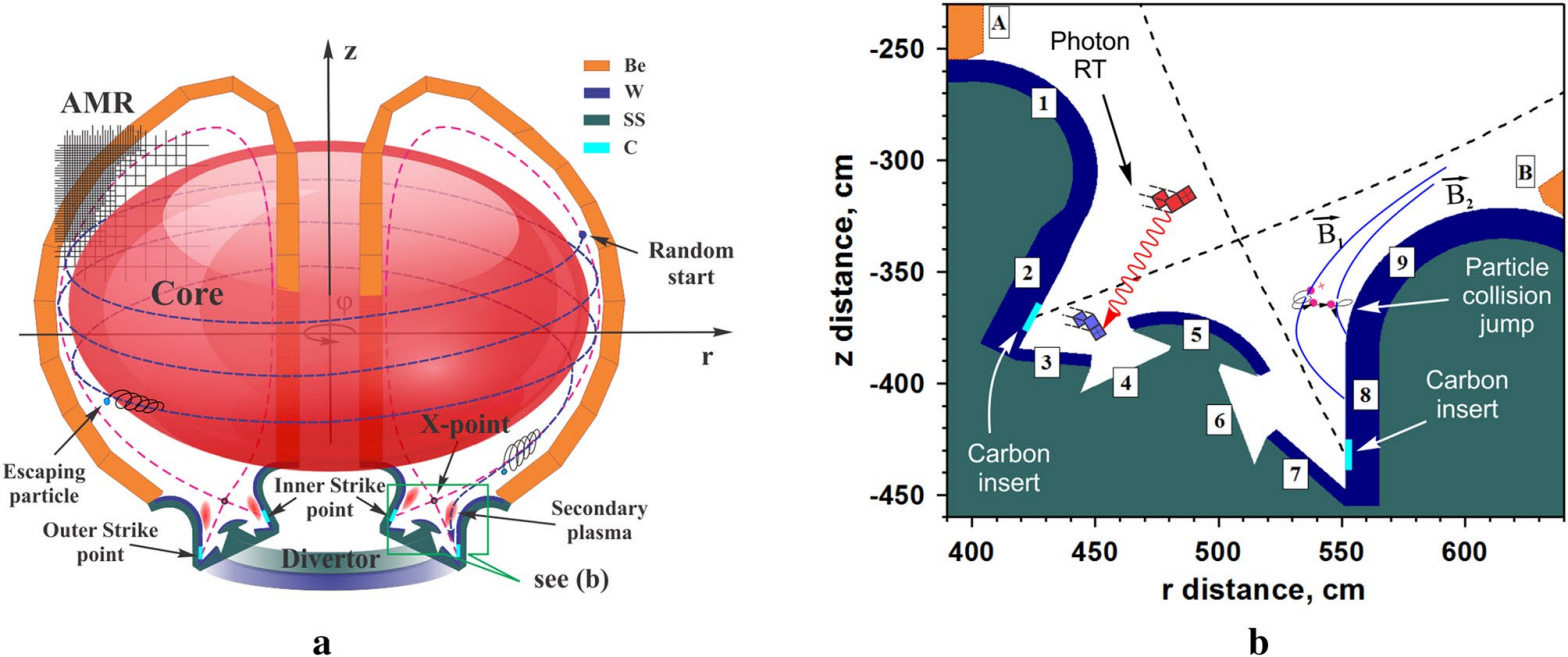
3D schematic illustration of simulating ITER device: coordinate system orientation, adaptive mesh refining (AMR) for component surfaces, wall materials palette (Be, W, SS), escaping core particles into divertor area (**a**); divertor area cross-section showing carbon insert location, initiation of secondary plasma, scattering of DT plasma particles with change of magnetic field lines, photon radiation transport (**b**). The images were prepared using CorelDRAW Graphics Suite 11.

The high-Z materials (e.g., tungsten) are very resistant to erosion as PFC but they are strong radiation emitters and thermonuclear reaction quencher when penetrated into the core plasma. The low-Z materials (e.g., carbon, lithium) are weak radiators and are more passive to cooling the core plasma but much less erosion resistant. Liquid metals are harder to implement in reactors, particularly in the current ITER device. Both theoretical and experimental estimations can accurately predict ITER power and particle fluxes on the divertor plate surfaces during transient events and loss of confinement^7,8^. These power/particle loads taken as the initial and boundary conditions in our simulations determine the response of divertor components, its vaporization, and plasma formation and expansion into the divertor space and SOL. The intensity and magnitude of initial vaporization, secondary plasma production, and expansion depends on the value of the power fluxes at the strike point area. These SP fluxes can be estimated by various methods. In the literature, good agreement exists for the total energy of disruption thermal quench (120–175 MJ) and correspondingly about 10% of this value is predicted for ELMs, i.e., up to 17.5 MJ^8,9^. Design of the ITER divertor went through several upgrades over the years starting from the original design concept^10^ to an advanced version^11^ and to the current geometry that we used in the present work^12^. We implemented the fine details of the geometry of the divertor and the chamber walls that can be found in review papers described the after-2007 design update^13,14^. We used ITER chosen materials, e.g., tungsten as the main divertor material, stainless steel as umbrella tubes material, and beryllium as chamber first wall material (see descriptions in Fig. 1). We assumed carbon inserts around the strike point location covering the footprint of ELMs and disruptions for simulation of low-Z secondary carbon plasma evolution.

We have developed an accurate comprehensive 3D Monte Carlo kinetic (MC) model^15^ and implemented into the HEIGHTS integrated code package. The MC kinetic model simulates the spatial and temporal power flux profiles of the divertor plates for various conditions (Fig. 1). The detailed simulation of the escaping core plasma particles into the SOL (Fig. 1a) was used in the integrated model as the input volumetric power source for the MHD module in HEIGHTS^5^.

The quadtree adaptive mesh refinement (AMR) algorithms is constructed using 5-level sublayers. We use such hierarchy levels for accurate simulation of the complex multiple geometries of ITER component and surfaces. These AMR algorithms include the entire divertor and SOL areas starting from few centimeters sizes, up to few millimeter sizes for MHD cells located near surfaces. For the subsurface processes, an additional refinement is used in various components with cell sizes less than one micron. The additional refinement allowed accurate simulation of the spatial energy deposition depth of penetrated particles into the surfaces and precise predictions of melt layer length and depth.

The integrated algorithms recalculate the particle flow every MHD time step starting from the last closed flux surface (LCFS) and finishing at the PFC surfaces. Each time step simulation requires ~ 10^3^ particles to accomplish an inexpensive accuracy of calculation within reasonable simulation time. For simulation of the 1 ms transient event duration, the MHD time step was assumed ~ 50 ps. As a result, the full simulation requires 2 × 10^7^ MHD time steps. The MC core particles escaping module recalculates the core plasma fluxes every 100 MHD time steps. The final count provides 2 × 10^5^ × 1000 = 2 × 10^8^ MC particles for the full simulation of one transient event. The integration of these particular solutions for each time step provides the solution of the general dynamic problem similar to our previously developed solutions for the heat conduction and magnetic diffusion problems^16^. Dynamically (every hydrodynamic time step) we recalculate plasma properties in each computational domain, update atomic plasma emission and absorption data, and calculate photon radiation transport (RT) and energy deposition in the evolving secondary plasma^17^. The fine details of the photon energy deposition of the divertor produced plasma on the surfaces of plasma facing and interior components determine the radiation time-dependent component of the heat loads and are predicted for the actual full design of ITER. The MC RT calculations were provided in full spectrum (from 0.05 to 10^5^ eV) in each cell with selection of ~ 2 × 10^4^ optical groups for a tungsten plasma and ~ 4 × 10^4^ for a carbon plasma. Every RT recalculation used up to 10^4^ quasi-photons per hydrodynamic cell (Fig. 1b) depending on the cell weight factors^15^. Therefore, the total number of particles used is 2 × 10^5^ × 10^4^ = 2 × 10^9^ particles for the full simulation of the secondary plasma radiation transport. The values chosen here were optimized for this study and are sufficient for accurate assessments in reasonable simulation time using supercomputer clusters. The previous knowledge of the escaped core plasma fluxes impact on the divertor plates is not sufficient for accurate prediction of the potential damage to the PFCs of various reactor components. To accurately assess the final surfaces response, erosion profiles, and components damage, the escaping core plasma model should be enhanced, efficiently coupled, and integrated with the evolving initial response of the divertor plate. The dynamic MC simulation of the incident DT plasma particles also includes numerous collisional processes with the evolving secondary plasma starting from the core surface exit locations and ending in the PFC walls.

Implementing details of the collisional/scattering processes and the proper interaction cross sections in strong magnetic fields provides the required energy deposition into the secondary plasma and the predicted scattered particle showers deposition and penetration into plasma-facing components (e.g., see illustration in Fig. 1b, where #1, #9 are Baffles; #2, #8 are Divertor Plates; #3, #7 are Reflectors; #4, #6 are Dome Tubes; and #5 is Dome. #A, #B are Beryllium walls). The solution of the heat conduction equation in PFC walls determines the thermal response, melting depth, and vaporization erosion in all ITER components. Initial estimates showed that the vaporization of divertor material, even in the case of 1.0 ms ELM event, is sufficiently large and as a result the divertor space will quickly be filled with tungsten or carbon vapor/plasma with a density much higher than the incident fuel plasma. Immediately after the establishment of the secondary plasma, the divertor plate surface will be shielded early in time during the event. The ongoing escaping core plasma will then mainly deposit its energy into the developing divertor vapor/plasma or can be scattered by vapor cloud to other surrounding locations.

Our recent modeling results for the initial magnetic equilibrium configuration in the ITER design showed that the maximum heat flux at the outboard SP to be 28.6 GW/m^2^ for disruption and 2.9 GW/m^2^ for an ELM. In this study, we focused our analysis on the most likely duration of an ELM or a disruption to be 1.0 ms as estimated in Refs.^7–9^. We assumed the total energy released during the ITER ELM is about 12.6 MJ that is 10% of the total energy contained in the core plasma. Our HEIGHTS simulations showed a tungsten plasma density of up to ~ 10^16^ cm^−3^ is developed near the original strike divertor surface that is much higher than the D/T core plasma value ~ 10^13^–10^14^ cm^−3^. As result, the mass added into the secondary plasma cloud from the escaped core DT plasma is negligibly small and has no major effects on the secondary plasma dynamics. The main input is the DT energy deposited into the secondary plasma during the secondary plasma expansion through the SOL. The DT energy deposition into this dense plasma cloud causes its motion and propagation as well as the motion and changes in the directions of the frozen magnetic field lines. The motion of the magnetic field lines will affect the DT plasma particles spatial interactions with the dense plasma and redirects the scattered particles showers to various internal locations away from the original SP location.

**Figure 2:**
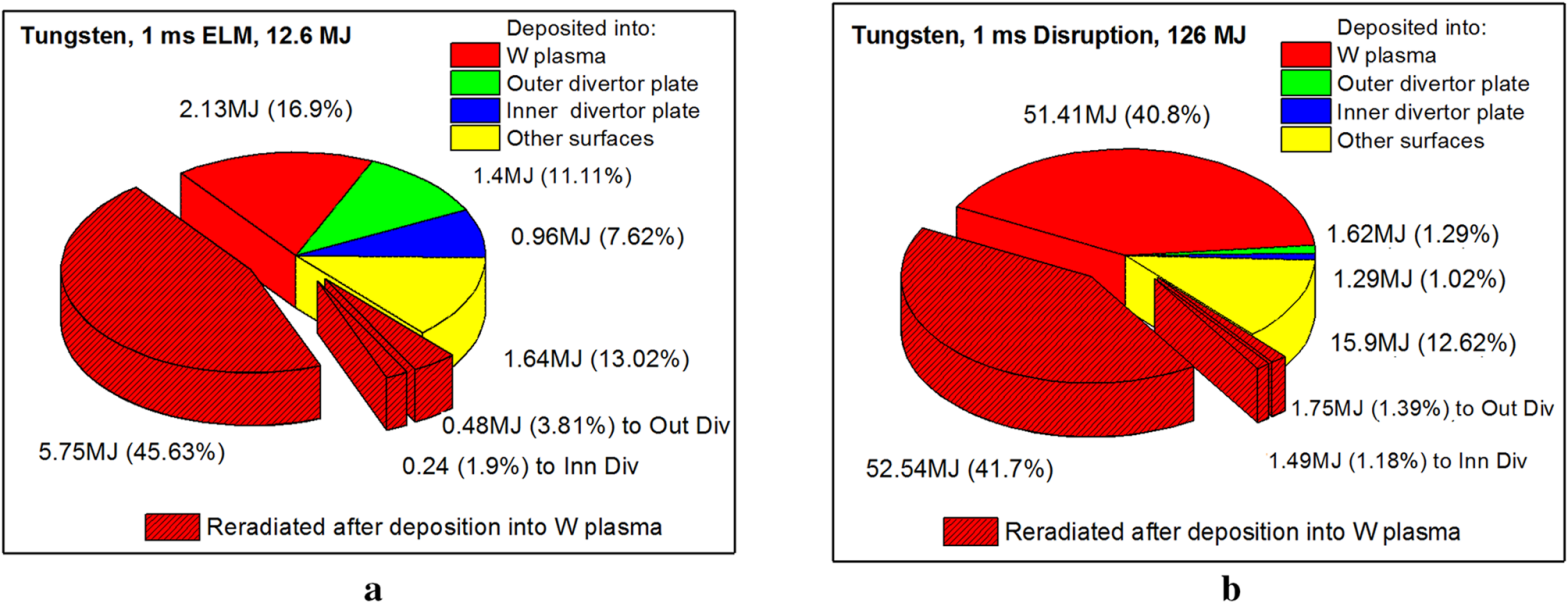
HEIGHTS predictions of the final energy balance in ITER transient events with tungsten SP: 1.0 ms ELM (**a**), and 1.0 ms disruption (**b**). The images were prepared using OriginPro V2020.

Opposing to the hydrogen isotope plasma, the low-temperature dense secondary plasma has much higher photon re-radiation power. The fine details and the RT of the divertor-produced plasma processes must be included and implemented into the full 3D real reactor modeling. This is very important and requires significant calculations load. The secondary plasma is then heated, ionized, excited, and as a result intensively reradiates photons. This photon transport in the produced dense plasma is not predicted or usually included into tokamak simulation codes^18,19^. We however, have developed extensive integrated MC models^5^ where we perform extensively detailed 3D photon transport within the secondary plasma. In this study we focused on tungsten material as chosen for ITER divertor plates and compare with our suggestion of including a thin carbon strip around the SP to absorb the initial energy of the ELM or disruption. Figure 2 shows the final energy distribution balance from the predicted initial parameters of ITER ELM and disruption events. In both cases, the evolved tungsten secondary plasma absorbs the majority of the incident core particles energy (red colored sectors), i.e., 68% for the ELM and 85% for the disruption event, regardless of the huge difference in the energy released. The quickly produced secondary plasma then effectively shields the divertor plate surface from the direct impact of the core plasma particles. The net energy deposited into the outer divertor plate has comparable values: 1.4 MJ for the ELM and 1.62 MJ for the disruption despite the large differences in the initial incident energy. The ELM is considered a regular event (~ 1 Hz)^9^ during ITER normal operation. In the case of the tungsten SP, more than 68% of the total ELM energy is contained in the highly radiative dense plasma. Severe damage to other nearby and hidden components can occur as well as other adverse effects due to potential core plasma contamination that could terminate each ELM into a disruption. In summary, the hydrodynamics and re-radiation processes of the secondary plasma evolution will determine the overall damage and consequences from these transient events.

The patterned red sectors (Fig. 2) show the emitted/reradiated photons energy from the secondary plasma. More than half (51%) of the total incident energy will be converted into photons during an ELM (Fig. 2a) and about 44% during a disruption (Fig. 2b). These numbers demonstrate that using the classical MHD equations without the secondary plasma photon deposition and transport significantly ignore up to half of the initial energy that is converted to photon radiation during transient events. One should also note that photon transport does not depend on the magnetic field and photons are free to go anywhere inside the chamber within the line of sight.

As shown in Fig. 2b, the photon energy deposition from the secondary plasma even into the shielded divertor plate can be larger than from the direct core plasma impact: 1.75 MJ photons and 1.62 MJ charged particles for the outer divertor plate. Below we analyze where such photons radiation energy goes and what damage it may cause to various internal components.

**Figure 3:**
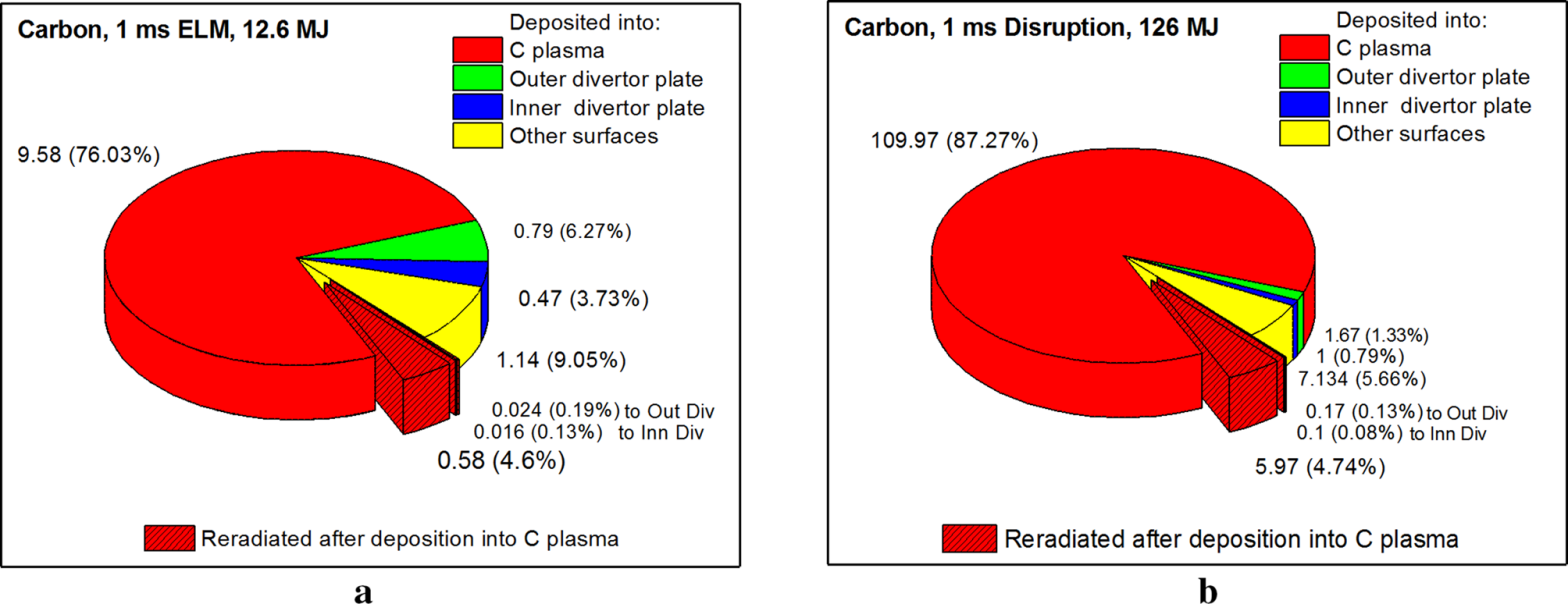
HEIGHTS predictions of final energy balance in ITER transient events with carbon insert at SP: 1.0 ms ELM (**a**), and 1.0 ms disruption (**b**). The images were prepared using OriginPro V2020.

Opposite to the high-Z tungsten plasma, the low-Z plasma has much lower capacity for photon radiation power due to its atomic structure. The carbon secondary plasma will absorb the core escaping particles energy mainly into the thermal component and not reradiate photons as tungsten. According to Fig. 3a, part of the total ELM energy (12.6 MJ) deposited into the carbon plasma increases up to 10.2 MJ (from 8.6 MJ for W). In addition, carbon plasma reradiates as photon energy only 0.62 MJ in comparison to 6.47 MJ for tungsten plasma. The photon radiation is highly difficult to mitigate and it's transfer time is very short. The plasma thermal energy transport is much slower that allows longer time for heat sink. Figure 3b shows similar behavior for the 1 ms disruption event: the photon radiation part significantly falls from 55.78 to 6.24 MJ when using thin carbon insert around the SP. The net reduction in photon energy part is ~ 90% for these transient events compared to all tungsten divertor design. Obviously, the secondary plasma evolution and dynamics contributes significantly to the final energy deposition in divertor surfaces. Below we present self-consistent simulations of the time-dependent damage sources.

## Full 3D dynamic simulation results

**Figure 4:**
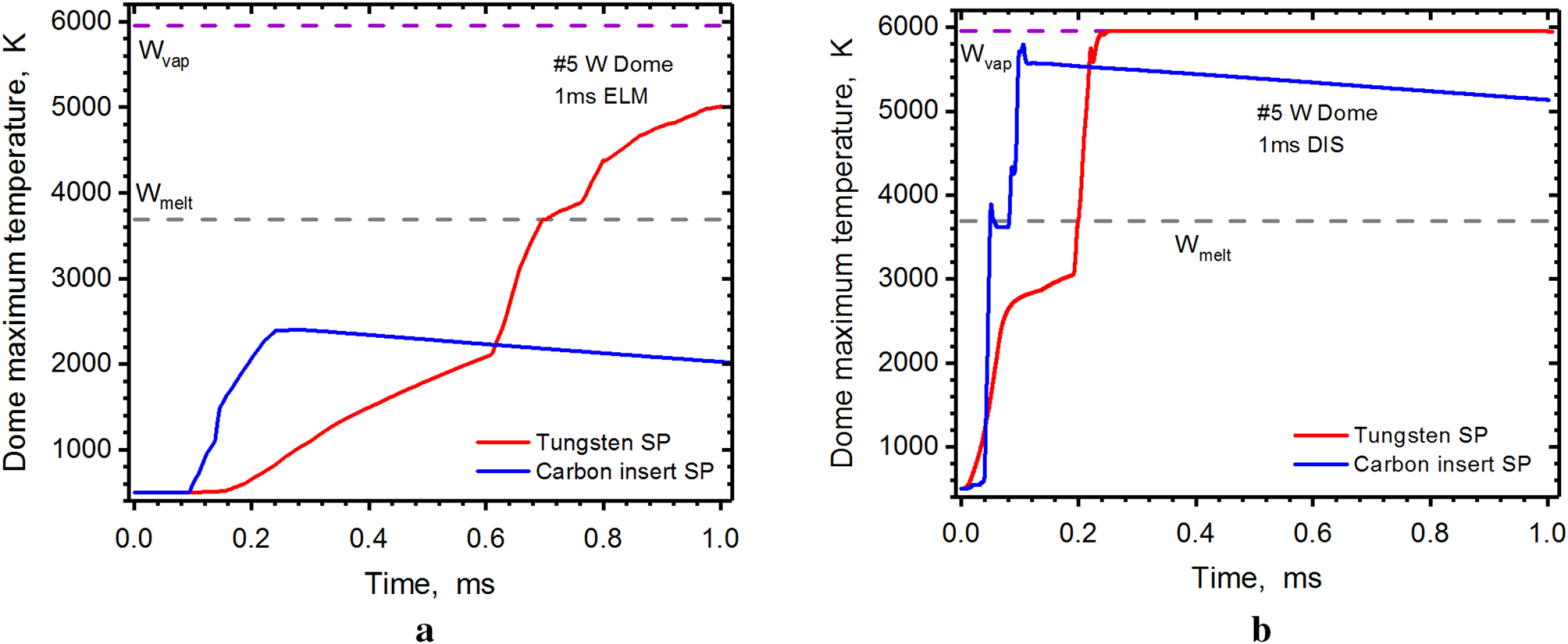
HEIGHTS simulation of PFC transient response: Dome maximum surface temperature during 1.0 ms ELM (**a**), 1.0 ms disruption (**b**), see Fig. 1b for surfaces location. Tungsten SP (red), carbon insert at SP (blue). The images were prepared using OriginPro V2020.

Our new efficient Monte Carlo algorithms can detect all incident particles (charged particles and photons) at any spatial point and time moment inside the entire ITER 3D device geometry and then calculate the energy fluxes (particles and photon radiation) from both the escaped core plasma and from the re-radiated photons from the developed secondary plasma. Recently, we reported about the critical heating spots on the tungsten divertor components during the ELMs and disruptions^6^. In this work, we simulated the same 1 ms ELM and disruption when using thin carbon insert within the tungsten divertor and compared with the pure tungsten heat loads simulations. Figure 4 shows the thermal response of the ITER dome due to a 1.0 ms event, which was originally thought to only damage the divertor plates. The surfaces and numbering of internal components are similar to those in our previous publication^5^ and are also shown in Fig. 1a. The surfaces response to such heat loads was calculated for all internal PFC. Despite that the radiation fluxes and tungsten plasma density during the ELM event are much smaller in comparison to a disruption, the simulations show melting spots on the dome surface up to ~ 15.0 µm deep and up to ~ 2.0 cm wide, which could be serious source of impurities. This melting spot appears due to insufficient secondary plasma shielding of the scattered particle showers of the incident core plasma in this area at the time t = 0.65 ms. This damage problem of the dome surface (#5) due to melting during the ELM^6^ will be fully avoided with installation of a carbon insert (Fig. 4a). This surface will not also be vaporized during the unmitigated disruption, as it shown in Fig. 4b.

**Figure 5:**
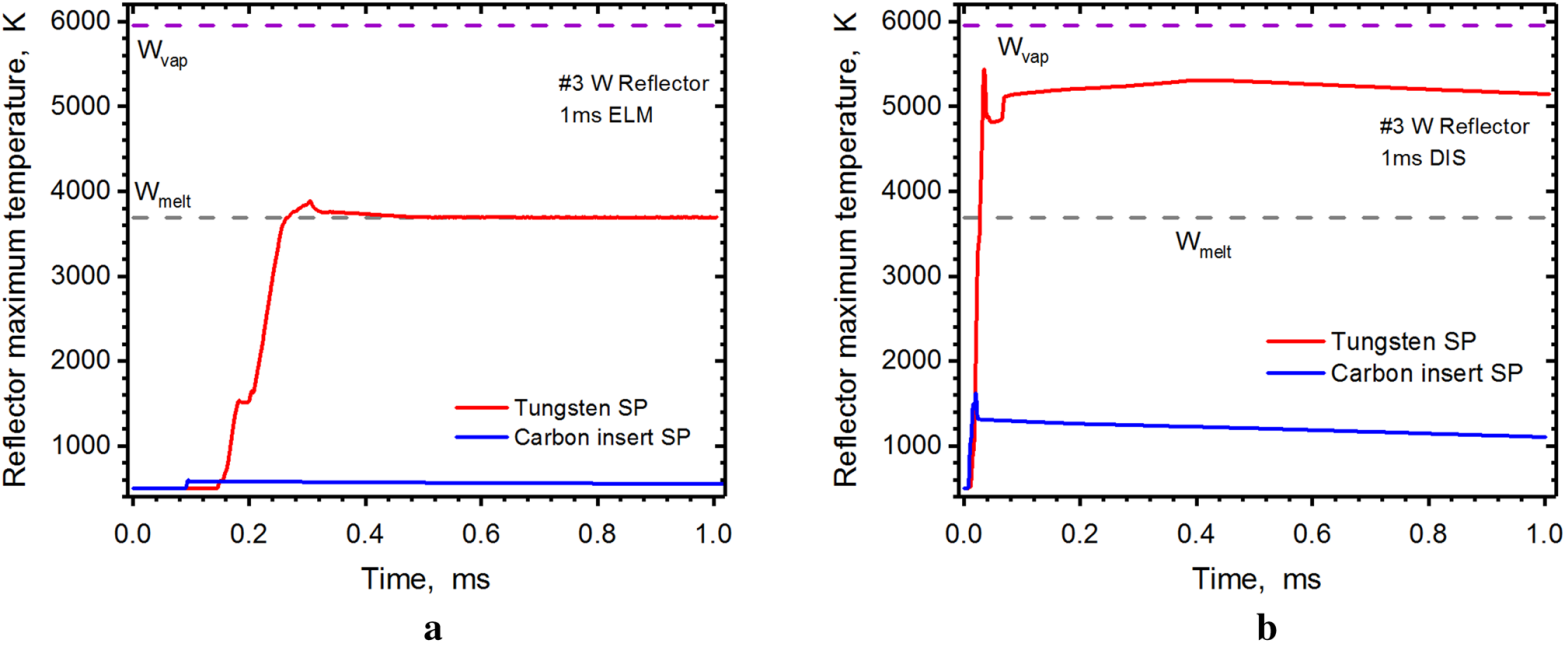
HEIGHTS simulation of PFC transient response: reflector maximum surface temperature during 1.0 ms ELM (**a**), 1.0 ms disruption (**b**), see Fig. 1b for surfaces location. Tungsten SP (red), carbon insert at SP (blue). The images were prepared using OriginPro V2020.

The reflector plates surfaces usually have expected and predictable heat loads and damage. It was previously believed that simple replacement of these plates every now and then will keep the reactor in good working condition. Figure 5 shows great decrease in heat load of reflector (#3) with the carbon insert. Even during the disruption, this reflector surface will not even melt (Fig. 5b).

**Figure 6:**
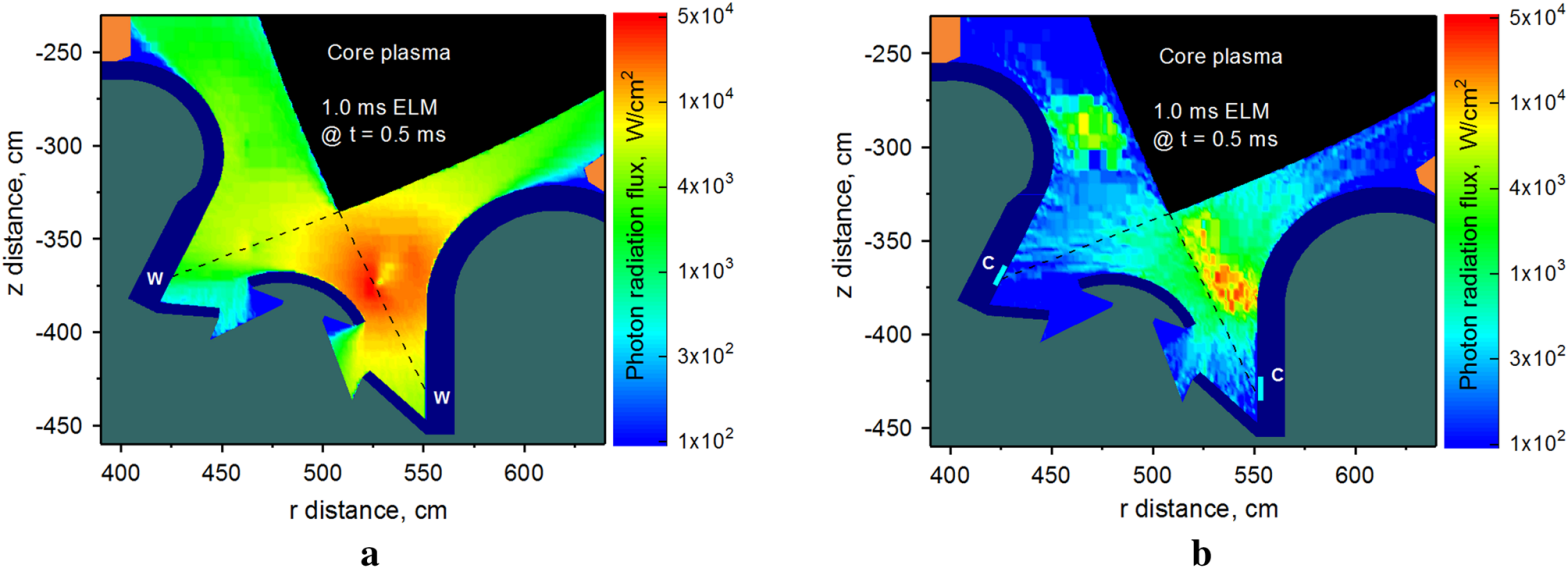
Snapshot of calculated photon radiation fluxes of secondary plasma at t = 0.5 ms during 1.0 ms ELM: tungsten SP (**a**), carbon insert at SP (**b**). The images were prepared using OriginPro V2020.

We should again note that the spots or the areas of the maximum temperatures are dynamic and time-dependent and move along the surfaces during the event duration due to the motion of the two main damage sources: core plasma scattered particles and photon radiation from the dynamically evolving secondary plasma. Figure 6 shows the calculated ELM photon radiation plots using the same logarithmic palette scale. The fluxes are plotted at the halftime moment (0.5 ms) of event duration. The tungsten radiation fluxes cover the entire divertor space and heat load practically all component surfaces (Fig. 6a) opposite to the local radiation "bulbs" when using carbon inserts on inner and outer divertors (Fig. 6b). The abrupt heating occurs as a result of the scattered particle showers of the incident core plasma from the front of the evolving dense secondary plasma. This damage effect is enhanced due to the additional continuous photon radiation of the secondary plasma at these locations as well. The intense photon radiation is localized in areas at the front of the secondary plasma clouds having higher temperatures. The DT core plasma deposits part of its kinetic energy at the front of the secondary plasma and is converted to photon radiation.

**Figure 7:**
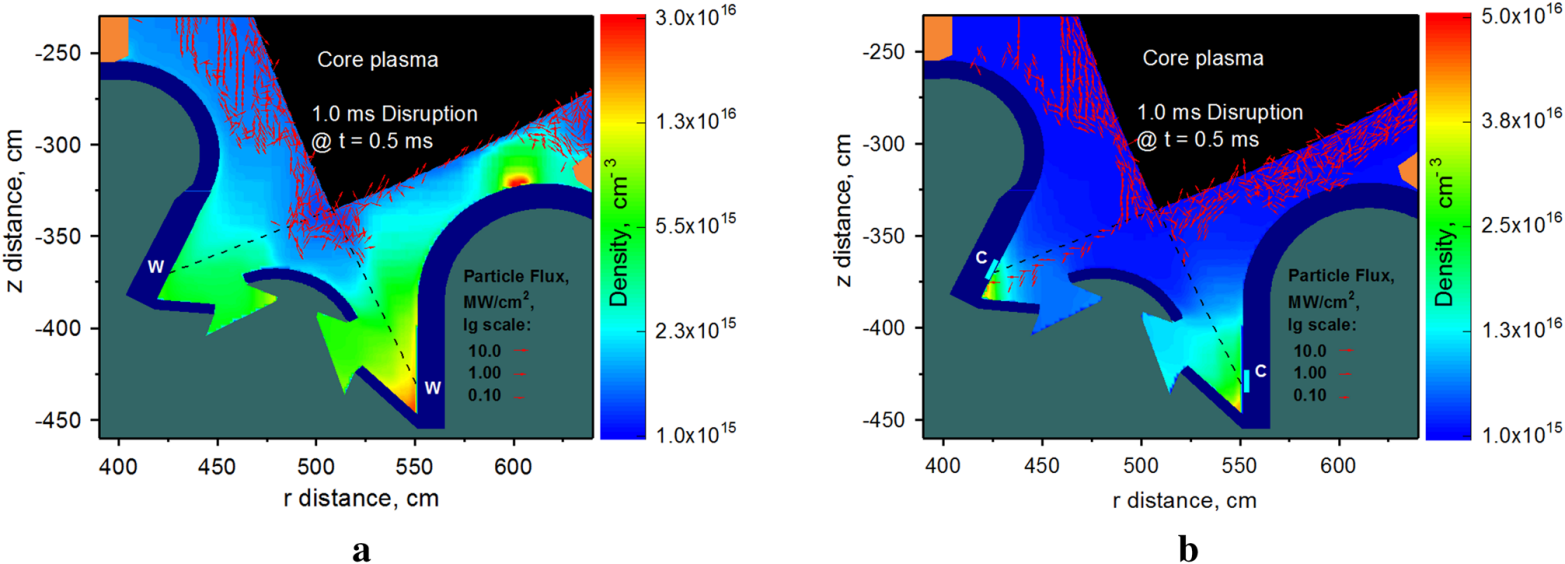
Snapshot of calculated of core plasma particle flux (vectors to scale) at t = 0.5 ms during 1.0 ms disruption on background of secondary plasma atomic density: tungsten SP (**a**), carbon insert at SP (**b**). The images were prepared using OriginPro V2020.

**Figure 8:**
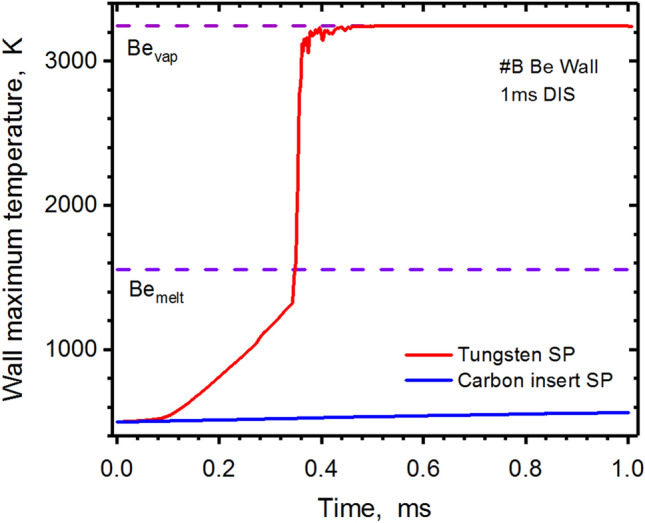
HEIGHTS simulation of PFC transient response: Beryllium wall (#B) surface maximum temperature during a 1.0 ms disruption, see Fig. 1b for surfaces location. Tungsten SP (red), carbon insert at SP (blue). The images were prepared using OriginPro V2020.

The scattered core plasma particles can over load the divertor components and initiate local vaporization spots as shown in Fig. 7a for the tungsten SP case. The tungsten dense gas/plasma cloud is a strong shield to the divertor plate and a dense scattering media for the continued incoming DT core plasma particles. The incident DT plasma particles deflect during the scattering processes with the dense secondary plasma and deposit their energy and cause new melting/vaporization spots on different locations as the dense secondary plasma propagate upward along the magnetic field lines in SOL. The scattered core plasma particles and the continuous dynamic photon radiation power cause the vaporizing spot on the Baffle surface (#9) near the beryllium first wall (#B). By using C inserts, one avoids vaporization as shown in Fig. 7b. As result, the active scattering of core escaping particles on this local cloud follows to the unexpected damage of the beryllium wall. The particle flux is plotted as vectors in logarithmic scale in Fig. 7 to clearly show the location and direction of the disruption impact. Figure 8 shows the result of carbon insert on the beryllium wall (#B) performance during disruption on the divertor plate. The C inserts offer complete protection of the first wall compared to significant melting and vaporization in the case of all tungsten divertor.

The effects of the secondary plasma evolution are dynamic and vary with the transient initial energy and its time characteristics. The damage front reaches the Be wall at the time t = 0.4 ms and will vaporize the Be first wall surface in the case of tungsten SP.

The above described heat loads and damage consequences are related to ITER operation during ELMs and unmitigated disruptions. The 1.0 ms ELM scenario expected at normal operation is more optimistic than a full unmitigated disruption and may not result in any vaporization spots on any nearby components other than the divertor plate but it could result on melting spots on the dome structure which may cause core plasma contamination. The erosion depth of the divertor plate predicted by our HEIGHTS simulation for the 1.0 ms ELM with tungsten SP is estimated as ~ 7.0 × 10^–3^ µm and is in agreement with published data^9^. However, the vaporization of the divertor plate and the subsequent generation of a secondary tungsten plasma soon after the start of the ELM event will have similar consequences as the disruption events. The amount of generated secondary divertor plasma during an ELM is still dense and hot enough to generate significant photon radiation fluxes similar to those predicted for disruptions.

**Figure 9:**
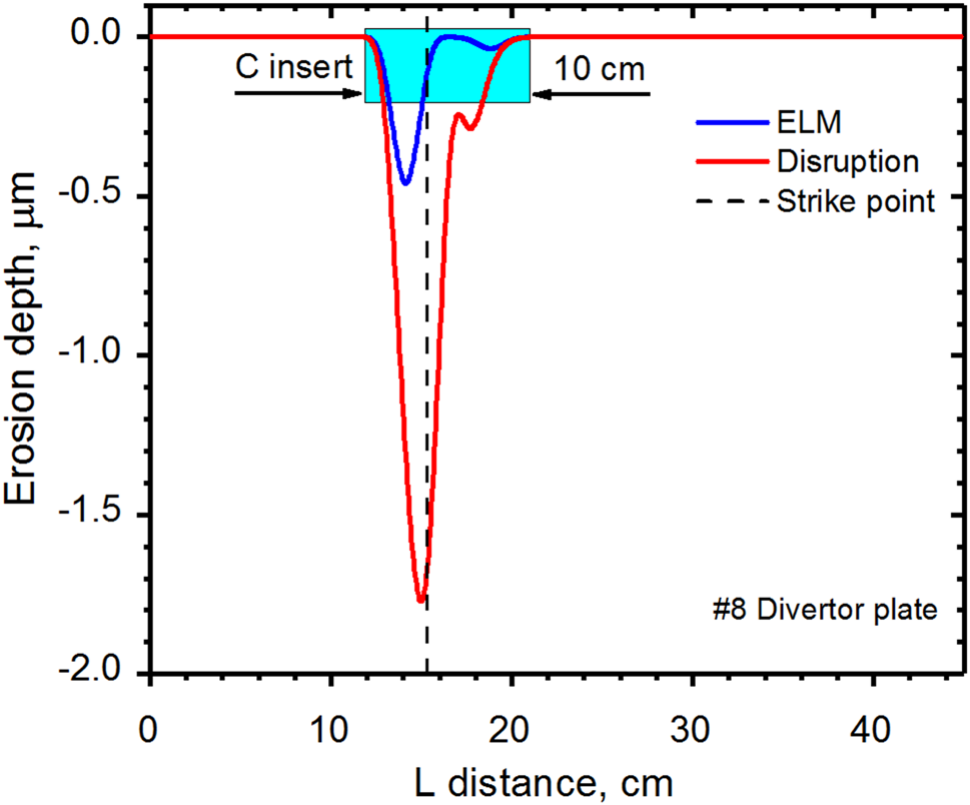
HEIGHTS simulation of divertor plate erosion crater and proposed carbon insert location. The images were prepared using OriginPro V2020.

Figure 9 shows the predicted damage crater on the divertor plate and the proposed size of the carbon insert on the tungsten plate at the SP during unmitigated transient events that sufficiently mitigates the damage of nearby components. The insert carbon strip is estimated to be ~ 10 cm width and can be assembled as interchangeable elements.

## Discussion

The comprehensive full 3-D integrated HEIGHTS simulations shows a critical need in the divertor required physics understanding when increasing the device power such that will generate a dense secondary plasma compared to current small low-power tokamaks. Normal operation in future ITER-like sizes will have ELMs core plasma fluxes high enough to form secondary divertor plasma at the strike points. The secondary plasma will require comprehensive and complex simulations and the current scaling scenarios developed for small tokamaks will not be valid. The coupling of the escaped core plasma interaction with the divertor produced plasma is critical in understanding the overall damage of the reactor plasma-facing and interior components during the transient events. The magnetohydrodynamics, optical plasma properties, and photon transport in such dense plasma are very complex processes and require careful simulation and proper physics. The HEIGHTS package is well benchmarked in these areas^20,21^.

As shown above, the concentration of predicted ELM and disruption fluxes in a narrow tungsten surface area in ITER design will unavoidably initiate and develop a highly radiative secondary high-Z plasma that transfer the initially expected transient heat loads on the divertor plate to other nearby and hidden components. The idea of simply repairing or replacing the whole divertor plate when sufficiently damaged is no longer the case. One can think of this behavior as “Conservation of Damage”. Dumping an intense energy in a small localized and closed area may protect the original divertor plate due to vapor/plasma shielding effects but the intense generated radiation and the scattered incident plasma particle fluxes from such dense plasma cloud will cause damage to other locations in such closed space. Several ways can be considered to mitigate this problem. For example, the divertor design can be modified to expand the strike point area in order to decrease the heat load here and to prevent formation of the high-Z secondary plasma. The “Snow-Flake” (SF) and “Super-X” (SX) magnetic configurations^22,23^ alternatives may offer a full solution or mitigation strategy to this problem. The magnetic field configurations can be used for distribution of plasma transient event power around a larger region on the divertor plate to decrease the surface heating. Relocating the strike points with the divertor plates remote from the core plasma is another possible solution to avoid excessive surfaces overheating^24,25^. Development a separate divertor chamber may also change this problem and could eliminate the certain high-Z plasma drift and penetration trough the LCFS reducing core plasma contamination^26^. The exploring solution is injection of low-Z neutral gas or pellet into the SP space during the event to protect the divertor plate and the nearby components utilizing the plasma shielding effect^27^. Liquid lithium can be used as PFC to mitigate the erosion problem and to improve core plasma stability and energy confinement. The implementation of a liquid lithium divertor, while may be a challenging task, is a very promising pathway to achieve reliable fusion energy production in the strong magnetic field confinement of tokamak reactors^28^.

In this work, we proposed and studied a mixed version of the divertor plate design for ITER device. We proposed to have a toroidal strip of small carbon inserts around the strike point (SP) but keeping the whole divertor design mainly as tungsten. The carbon insert was proposed to take advantage of the low radiation properties of low-Z materials to prevent significant damage to nearby and hidden components during ELMs and disruptions. While carbon has several disadvantages as plasma facing or divertor materials and was extensively studied in the past, the proposed use of carbon in this study is for a very limited amount that can be easily repaired or replaced but could be very critical in mitigating the serious effects of plasma transient events. This carbon insert acts similar to a source of low-Z gas injection or similar to the pellet injection in order to prevent significant damages during transient events or can easily repair local components instead of damages to sensitive internal components that would require extensive repair time.

## Conclusion

A key obstacle to a successful magnetic fusion energy production concept is performance during abnormal events including plasma disruptions and edge-localized modes (ELMs) due to loss of plasma confinement. Any credible reactor design must tolerate a few of these transient events without serious consequences. The comprehensive HEIGHTS simulation package has expanded capabilities in simulating all integrated physics in full 3D of any tokamak real geometry and carefully evaluating components response during transient events and assessing potential damage mechanisms to every internal component of the cassette structure as well as the first walls. The package is focused on the detail formation of the secondary plasma generation, evolution, propagation through the SOL and its interaction with the incoming disrupting D/T plasma and their effects on divertor component and nearby surfaces. The expected increase in future tokamak power, i.e., ITER and beyond, will increase the power flux on the divertor plates during transient events beyond a critical value that causes surface vaporization and initiation of a secondary plasma from the divertor materials. The generated secondary plasma will be evolving around the strike point, intensifying, and intercepting the remaining incoming transient disrupting and ELM particles, expanding into the SOL, and in case of ELMs potentially penetrating into the core plasma with possible termination of the discharge into a full disruption following each ELM. The disrupting plasma particles energy is basically converted into two significant secondary heat sources that can cause serious damage to internal and hidden components that were not directly exposed to disruptions and ELMs through photons and particles transport and deposition. The collisions of the incident core particles with the secondary plasma and the deflected/scattered energetic particles under the strong modified magnetic field structure will lead to significant energy transfer to nearby component surfaces. To mitigate the secondary plasma effects, one should try to increase the initial core plasma impact area along the divertor plate, i.e., the expanding the "wet area", or to place the divertor plates away from the tokamak core, or to divert disrupting and ELM plasma into a larger separate compartment. Another option is to develop a "detached pumped" divertor chamber. This will require significant design changes.

We simulated the response due to unmitigated transient events when using a small toroidal carbon insert around the strike point area to avoid the expected high-Z tungsten secondary plasma formation and its intense radiation compared with carbon low-Z plasma during transient events. During transients, most of the damage to nearby components that are hard to repair is resulting from the intense radiation of the secondary generated divertor plasma. This carbon insert acts similar to a source of low-Z gas injection or pellet injection to protect significant damage during transient events and allow easily repair of divertor components instead of the damages expected from pure tungsten divertor to many sensitive interior components. Our simulations showed that using the small carbon insert with size of about 10 cm around the strike point (SP) can significantly reduce the overall expected damage on the tungsten dome structure, reflector plates, even the Be first walls and prevent tungsten vaporization and its potential high-Z core plasma contamination.

## Methods

Methods details, including statements of data availability and any associated accession codes and references, are also available at 10.1038/s41598-021-81510-2.

### Simulations setup

HEIGHTS simulation package is developed over the last three decades to study the effects of any form of intense energy or power deposition on target materials and components for various energy, industrial, and defense applications. We have extensively upgraded HEIGHTS to study and simulate the effects of transient plasma events in magnetic fusion devices such as ITER and future DEMO machines. The initial simulation conditions for ITER device included three main parameters: (1) pedestal energy *W*_*ped*_ = 126 MJ, (2) temperature of pedestal *T*_*ped*_ = 3.5 keV, and (3) transient event duration *τ* = 0.1–3.0 ms to cover all possible transient duration range. We used a total of 126 MJ for disruption and 12.6 MJ for giant ELM. We varied the event duration in the expected range of 0.1–3.0 ms but in this study we only focused on the most anticipated duration of 1.0 ms. The initial conditions of the equilibrium magnetic plasma configuration used in our study was obtained from the EQDSK database files^29^. We simulated the initiated divertor plasma evolution and its atomic physics, opacity, and radiation characteristics during and after an ELM or a disruption in ITER current exact three dimensional design^30^. Details of the ITER design implementation on an adaptive mesh are described in Ref.^5^.

The Bebop cluster at the Argonne National Laboratory^31^ was used for our simulations.

### Magnetohydrodynamics of the secondary plasma

The integrated models and packages were constructed in the 3D coordinate system where the secondary plasma MHD equations set is used as the model core^5,15^. The equations system describes the convective motion of the secondary plasma and should be coupled with the dissipative physical processes such as the heat conduction, radiation transport, magnetic diffusion, and the core plasma energy deposition during the transient events.

### Magnetic diffusion and plasma heat conduction

The magnetic diffusion equations have parabolic type and for its solution we used an implicit method based on sparse matrix solution. The solution determines dissipative changes of magnetic field that should be used for the correction of the MHD. The details of magnetic diffusion and plasma heat conduction in the evolving secondary plasma were extensively developed in Ref.^16^. We consider the dense secondary plasma here assuming transport coefficients studied in Refs.^32–35^.

### Kinetic Monte Carlo simulation of escaping core plasma particles

The model of core plasma particles evolution includes eight main scattering processes: ion-nuclear interactions, ion–electron interaction, electron-nuclear interaction, electron–electron interaction, Bremsstrahlung process, Compton processes, photoabsorption, and Auger recombination. The approximation of binary or pair collisions^36^ is used for simulations.

### Radiation transport and opacities of secondary plasma

Our recent integrated model^5^, was enhanced to allow the escaped core particles deposit part of their energy in the evolved vapor and then the secondary plasma during the collisions. For detail calculation of the secondary radiation, we developed a comprehensive Monte Carlo radiation transport (RT) model based on several weighted factors to significantly reduce the computational time^17,37^. The implemented weight factors in our MC model greatly reduced RT calculations compared to both MC models without weight factors and the direct RT solution methods^38^.

## Data Availability

The data that support the findings of this study are stored on Purdue Servers and on Argonne National Laboratory Bebop cluster and are available from the corresponding authors upon reasonable request.
